# No Time to Relax and Unwind: Exploration of Topoisomerases and a Growing Field of Study

**DOI:** 10.3390/ijms241713080

**Published:** 2023-08-23

**Authors:** Joseph E. Deweese, Neil Osheroff

**Affiliations:** 1 Department of Biological, Physical, and Human Sciences, Freed-Hardeman University, Henderson, TN 38340, USA; 2 Department of Biochemistry, Vanderbilt University School of Medicine, Nashville, TN 37232, USA; neil.osheroff@vanderbilt.edu; 3 Department of Medicine (Hematology/Oncology), Vanderbilt University School of Medicine, Nashville, TN 37232, USA

With the topoisomerase field in its sixth decade [1], it is worth taking a moment to pause and consider where our knowledge has come from and where the field is headed. From the time when topoisomerases were first studied in prokaryotes to their exploration in eukaryotic systems such as yeast and *Drosophila*, the field has advanced significantly, with dozens of laboratory groups and hundreds of researchers worldwide [2,3,4]. The pioneering work in yeast and other model systems laid a critical foundation for the research now performed with human enzymes, cells, and disease states. Additionally, the examination of prokaryotic enzymes has continued to develop, enabling the exploration and discovery of additional enzymes and antibacterial compounds that can be used to manipulate these enzymes and cure infectious diseases [5,6,7,8]. With the approval of antibody–drug conjugates targeting topoisomerase I, there is renewed interest in identifying novel compounds and understanding the mechanisms of topoisomerase-targeted anticancer agents [9]. Work on topoisomerases has transitioned from examinations in cellular systems to studies of purified enzymes with ensemble approaches to single-molecule experiments and structural studies that allow for the detailed analysis of the mechanisms and forces involved in topoisomerase catalysis [10]. Finally, studies that use single-molecule or genomic approaches have moved back into cells in order to detail the interactions and functions of topoisomerases in living systems [10].

Several themes in the field have emerged in recent decades. First, projects are becoming increasingly interdisciplinary in nature. This truism has occurred across science, and is certainly reflected in the topoisomerase field. For instance, we can see interdisciplinary collaborations aiding in the development of novel antibacterial and anticancer agents [11,12]. From studying purified enzymes to exploration with cellular models to applications in animal models, numerous areas of expertise are needed, often requiring collaborative efforts among multiple laboratory groups.

Second, with the increased study of genome function, critical roles of topoisomerases in maintaining topology and influencing genome regulation have been revealed [13]. These studies have also required expertise in multiple fields of study to explore and integrate topoisomerase function with genomic regulation. Interdisciplinary collaborations have enabled the mapping of topoisomerase binding sites across the genome and at different stages of the cell cycle in multiple model systems [14]. This wealth of information is incredibly valuable, but our ultimate comprehensive understanding of the regulation and operation of topoisomerases will depend on our ability to collate huge volumes of information.

Third, the rise of “data science” and bioinformatics has enabled the storage and processing of vast amounts of data (such as those mentioned above). Methods such as Hi-C that helped map interactions between and within chromosomes could be combined with topoisomerase binding maps to provide a more genome-wide vision of topoisomerase function [15]. Additional databases could also be brought into play, such as those that document post-translational modifications (e.g., Phosphosite), protein binding partners (e.g., The BIOGrid), and tissue-specific expression (e.g., Human Protein Atlas) [16,17,18]. We live and work in an era that provides huge quantities of data, and much of these data are freely available to the public. Accessing and interpreting data from these repositories often requires training or expertise that may require collaborative efforts. Alternatively, we may need to do a better job of training our current students and postdoctoral fellows in these areas.

Fourth, the integration of data from various sources has led to several studies that explore unknown or underappreciated aspects of topoisomerase function [19]. For instance, insights into the inter-relationships in the unstructured C-terminal domain may be explored using computational methods [20]. Granted, not all computational data are created equal. However, at the very least, there is value in the application of computational data to develop testable models for topoisomerase function and regulation.

What can we learn from the above observations? An obvious lesson is that we are stronger together rather than in siloed laboratories. The more we interact and collaborate, the more likely we are to make meaningful discoveries and advance the topoisomerase field. Because the critical roles of topoisomerases in cellular and developmental systems are coupled with their roles in disease development and therapy, new discoveries have the potential to influence our understanding of science and enhance human health. Enhanced interactions between colleagues and collaborators are supported by the increased use of video conferencing that began during the COVID-19 pandemic. Virtual discussions between researchers from around the globe now take place on a routine basis and will help us to keep in contact with one another.

Another important lesson is that we need to broaden the training of our students and postdoctoral fellows. Gone are the days when learning traditional enzymological methods and cell culturing techniques can suffice. Although these skills are still invaluable and continue to be utilized, the modern student needs to be exposed to bioinformatics, 3D genomics, and other resources and skill sets. The good news is that training in these areas is not hard to find. Whether formalized or through streaming videos, resources abound for training in these areas. Enabling students and postdocs to expand their training will foster a stronger scientific community and likely increase the collaborative nature of science in the topoisomerase and DNA topology fields. While newer methods and approaches must become a part of the training of our students, we should not lose sight of the detailed, careful enzymology experiments that have provided the basis for our current understanding of these enzymes.

Finally, we need to embrace the era of “data science” and find ways to leverage new tools to gain a deeper understanding of topoisomerase structure, function, and regulation. Utilizing data generated with these tools, we can design experiments that will help to validate and expand our knowledge of these fascinating enzymes. Once again, we will require collaboration and interdisciplinary interactions to accomplish such goals.

The topoisomerase field continues to expand and blossom in new and unexpected ways and will continue to do so if researchers incorporate new approaches, ideas, and data into their analyses. Here’s to the next 60 years of topoisomerase research!
